# Association of Intensive Endoscopic Screening Burden With Gastric Cancer Detection

**DOI:** 10.1001/jamanetworkopen.2020.32542

**Published:** 2021-01-07

**Authors:** Choong-Kyun Noh, Eunyoung Lee, Gil Ho Lee, Joon Koo Kang, Sun Gyo Lim, Bumhee Park, Jae Bum Park, Sung Jae Shin, Jae Youn Cheong, Jin Hong Kim, Kee Myung Lee

**Affiliations:** 1 Department of Gastroenterology, Ajou University School of Medicine, Suwon, Republic of Korea; 2 Stomach Cancer Center, Ajou University Hospital, Suwon, Republic of Korea; 3 Department of Biomedical Informatics, Ajou University School of Medicine, Suwon, Republic of Korea; 4 Office of Biostatistics, Ajou Research Institute for Innovative Medicine, Ajou University Medical Center, Suwon, Republic of Korea; 5 Department of Medical Sciences, Biomedical Informatics, Graduate School of Ajou University, Suwon, Republic of Korea; 6 Department of Occupational and Environmental Medicine, Ajou University School of Medicine, Suwon, Republic of Korea

## Abstract

**Importance:**

The rapidly increasing number of gastric cancer examinations performed over a short period might influence screening performance. Accessing the association between calendar month and gastric cancer detection rates might improve policy and guide institutional support.

**Objective:**

To evaluate the association between the increased number of examinations over a certain period and gastric cancer detection rates among a large population included in the Korean National Cancer Screening Program (KNCSP).

**Design, Setting, and Participants:**

This retrospective, population-based cohort study used data from the KNCSP comprising 26 765 665 men and women aged 40 years or older who participated in the screening program between January 1, 2013, and December 31, 2016. Data were analyzed from November 1, 2019, to March 31, 2020.

**Exposures:**

Gastric cancer screening with endoscopy.

**Main Outcomes and Measures:**

The primary outcome was monthly gastric cancer detection rates in the KNCSP. A negative binomial regression model was used to evaluate the association between the screening month and detection rates.

**Results:**

In total, 21 535 222 individuals underwent endoscopy (mean [SD] age, 55.61 [10.61] years; 11 761 709 women [54.62%]). The quarterly number of participants was the highest in the last quarter of the study period (2013-2014: 4 094 951 [41.39%], 2015-2016: 4 911 629 [42.19%]); this proportion was 2.48 to 2.84 times greater than that of the first quarter. Cancer detection rates were the lowest in December (2013-2014: 0.22; 95% CI, 0.22-0.23; 2015-2016: 0.21; 95% CI, 0.21-0.22); this was approximately a 40.0% to 45.0% reduction compared with the rates in January. The age group was the significant factor for monthly detection rates. After adjustment for the age group and taking account of the number of screenings, the estimated coefficient range for the screening month was negative and the detection rate in December was significantly different than in January for both the consequent cycles (2013-2014: −0.05 to −0.18; *P* < .001; and 2015-2016: −0.06 to −0.19; *P* < .001). In the multivariable logistic model, the association of calendar month with detected cancer remained after adjusting for other confounding factors (December, 2013-2014: odds ratio, 0.82; 95% CI, 0.76-0.87; *P* < .001; 2015-2016: odds ratio, 0.83; 95% CI, 0.79-0.89; *P* < .001).

**Conclusions and Relevance:**

The findings of this cohort study suggest that the workload of endoscopists increased with the increasing number of examinations toward the end of the year, as demonstrated by the decreased cancer detection rates. These findings may help to improve gastric cancer detection rates of screening programs by controlling the monthly screening number and policy modifications.

## Key Points

**Question:**

Is monthly variation in gastric cancer screening rates associated with changes in rates of cancer detection?

**Findings:**

This cohort study of 21 535 222 participants from the Korean National Cancer Screening Program database found that the monthly number of endoscopic examinations increased toward the end of the year. However, the gastric cancer detection rates were negatively and significantly associated with calendar month.

**Meaning:**

The findings of this cohort study suggest the importance of the implementation of policy guidelines to strengthen the merits of the screening program, including a reduction in gastric cancer-related mortality.

## Teaser

This cohort study compares the numbers of endoscopic examinations for gastric cancer screening over quarters of a calendar year with rates of gastric cancer diagnosed during those periods.

## Introduction

Although the prevalence of gastric cancer is decreasing, it remains the third leading cause of cancer mortality worldwide.^1,2^ Early detection, diagnosis, and treatment are the only methods to reduce mortality associated with gastric cancer. Diagnostic tools for early gastric cancer detection include upper gastrointestinal (UGI) series and endoscopy; previous studies have reported the superiority of upper endoscopy over UGI series for gastric cancer diagnosis.^3,4,5^

Some Asian countries, including Korea, conduct endoscopic screening programs for gastric cancer.^6^ These screening programs may reduce the risk of death from gastric cancer.^7^ In Korea, the Korean National Cancer Screening Program (KNCSP) for gastric cancer was launched in 1999 to provide gastric cancer screening via upper endoscopy or UGI series for individuals aged 40 years or older.^8^ The total number of participants in the KNCSP for gastric cancer surveyed from 2002 to 2018 (except 2012) was 71 773 605.^9,10,11,12,13,14,15^ The individuals enrolled in the KNCSP are required to have their examinations completed by December 31 of the corresponding year or they may lose their opportunity to be examined the following year. Because most people wish to delay their examinations until the latest possible time, the number of individuals undergoing examinations might vary monthly. Furthermore, because endoscopic screening has better diagnostic accuracy than UGI series,^3,4^ the number of individuals undergoing endoscopy has been increasing substantially. Consequently, endoscopists’ workloads are being steadily increased.

Several studies have shown that operator workload affects examination quality.^16,17,18,19,20,21^ Especially in index colonoscopy, adenoma detection rates decrease with increasing procedural hours in an endoscopist’s workload.^19^ We hypothesized that an increased operator workload toward the end of the year in the KNCSP would affect the quality of the screening program for gastric cancer. Controlling or limiting the number of endoscopic examinations performed in a screening program is a modifiable factor that might help to reduce gastric cancer–related mortality. In the KNCSP, a large portion of examinations was performed at the end of the year; to date, this issue has not been well described.

To our knowledge, this is the first study to use a large population database to assess the association between the increased number of examinations over a specified period and cancer detection rates in the KNCSP. In addition, we aimed to investigate the risk factors associated with the detection of gastric cancer at screening.

## Methods

### Study Design and Population

We conducted a retrospective, large, population-based cohort study between January 1, 2013, and December 31, 2016, using the KNCSP database after permission was obtained from the Ministry of Health and Welfare. Data were analyzed from November 1, 2019, to March 31, 2020. The total cohort comprised 26 765 665 men and women aged 40 years or older who participated in the screening program. The included participants were linked with the National Health Insurance Sharing Service–National Health Information Database (NHIS-NHID) to review their medical records until 2018. Participants were excluded if they had received previous gastric cancer diagnoses, had upper endoscopy performed for 2 consecutive years, and were not eligible for KNCSP. The study protocol was approved by the Ajou University Hospital Institutional Review Board and Ethics Committee and the requirement for informed patient consent was waived owing to the use of a deidentified data set for the analyses. This study followed the Strengthening the Reporting of Observational Studies in Epidemiology (STROBE) reporting guideline for cohort studies.

### Korean National Cancer Screening Program

The KNCSP and Korea Central Cancer Registry provide targeted populations (eMethods in the Supplement) with screening services for 6 common cancers: stomach, liver, colorectum, breast, uterine cervix, and lung. For gastric cancer, the screening frequency is every 2 years, determined by the individual’s birth year. The KNCSP recommends biennial screening with either UGI series or upper endoscopy for men and women aged 40 years or older who are eligible for the KNCSP every other year. Participants are required to undergo a screening test during their designated year any time from January 1 to December 31. The participants to be examined in the relevant year visit the examination institute (eMethods and eTable 1 in the Supplement) and complete a cancer screening questionnaire and undergo an examination. On completion of the questionnaire, the data are coded and stored in the database. If gastric cancer is suspected upon endoscopic examination, biopsy sampling is performed. The specific screening endoscopy protocol is described in the eMethods in the Supplement. When a histologic diagnosis of gastric cancer is made, the findings are confirmed and recorded in the national cancer registry.

### Data Collection and Definitions

The KNCSP data include a history of cancer screening, medical and family histories based on questionnaires, information from screening sites (region and hospital type) and screening providers, and cancer screening results. The data were separated based on the screening cycles to validate the consistency of the results found in the selected screening cycle: the first and second screening cycles were 2013-2014 and 2015-2016.

In this analysis, the screening results were defined as positive if the endoscopic results were recorded as possible gastric cancer, early gastric cancer, or advanced gastric cancer or the biopsy results were recorded as low-grade dysplasia, high-grade dysplasia, suspicious gastric cancer, or gastric cancer. Subsequently, we linked medical records from the NHIS-NHID until December 2018 and investigated medical records to detect interval cancer. Using medical records in the NHIS-NHID, detected cancer was defined as patients with positive screening results confirmed with a diagnosis code of gastric cancer (*International Statistical Classification of Diseases, Tenth Revision*, code C16.xx). Interval cancer was defined as participants with negative screening results who received at least 1 diagnosis code of gastric cancer (C16.xx) in the primary diagnosis within 1 year of the negative screening result.

### Statistical Analysis

We summarized the demographic characteristics of participants and the screening time and places using descriptive statistics and inspected trends in the number of participants based on month. Overall sensitivity, specificity, positive predictive value, detection rates per 100 positive screenings, interval cancer rates per 1000 negative screenings, and positive rates per 1000 screenings for each screening cycle were calculated. To explore monthly detection rates, we first investigated whether the number of participants varied from January to December and then calculated monthly detection rates, further stratifying the data based on age grouping.

To identify overall trends in detection rates, we applied a Poisson regression model first; however, the overdispersion problem was encountered. Thus, a negative binomial regression model was fit for the rate outcomes to investigate whether there was any association between the detection rates and screening month. Stratified analyses taking into account the age grouping were also performed. The number of screenings per month was included as an offset term and adjusted in the model. Overdispersion was tested using scaled Pearson χ^2^ statistics. Moreover, we evaluated the risk factors for cancer detection using a logistic regression. The covariance matrix was multiplied by a factor of deviance/degree of freedom. The univariate logistic regression model was first applied to choose the appropriate model for each explanatory variable: the history of endoscopy; sex, age group, screening month, hospital type, and metropolitan area; and history of atrophic gastritis, ulcer, intestinal metaplasia, gastric polyp, and other gastric diseases. Then, using the stepwise selection process at a significance level of .05 for the χ^2^ value for entering a variable into the model and a significance level of .05 for the Wald χ^2^ value for a variable to stay in the model, a multivariable logistic regression analysis was performed to assess the adjusted risk factors. Detailed statistical methods are provided in eAppendix 1 and eAppendix 2 in the Supplement. Odds ratios and 95% CIs for cancer detection were obtained. All reported *P* values were 2-sided, and *P* < .05 was considered statistically significant. Analyses were performed using SAS, version 9.4 (SAS Institute Inc).

## Results

### Study Population

**Figure 1.**
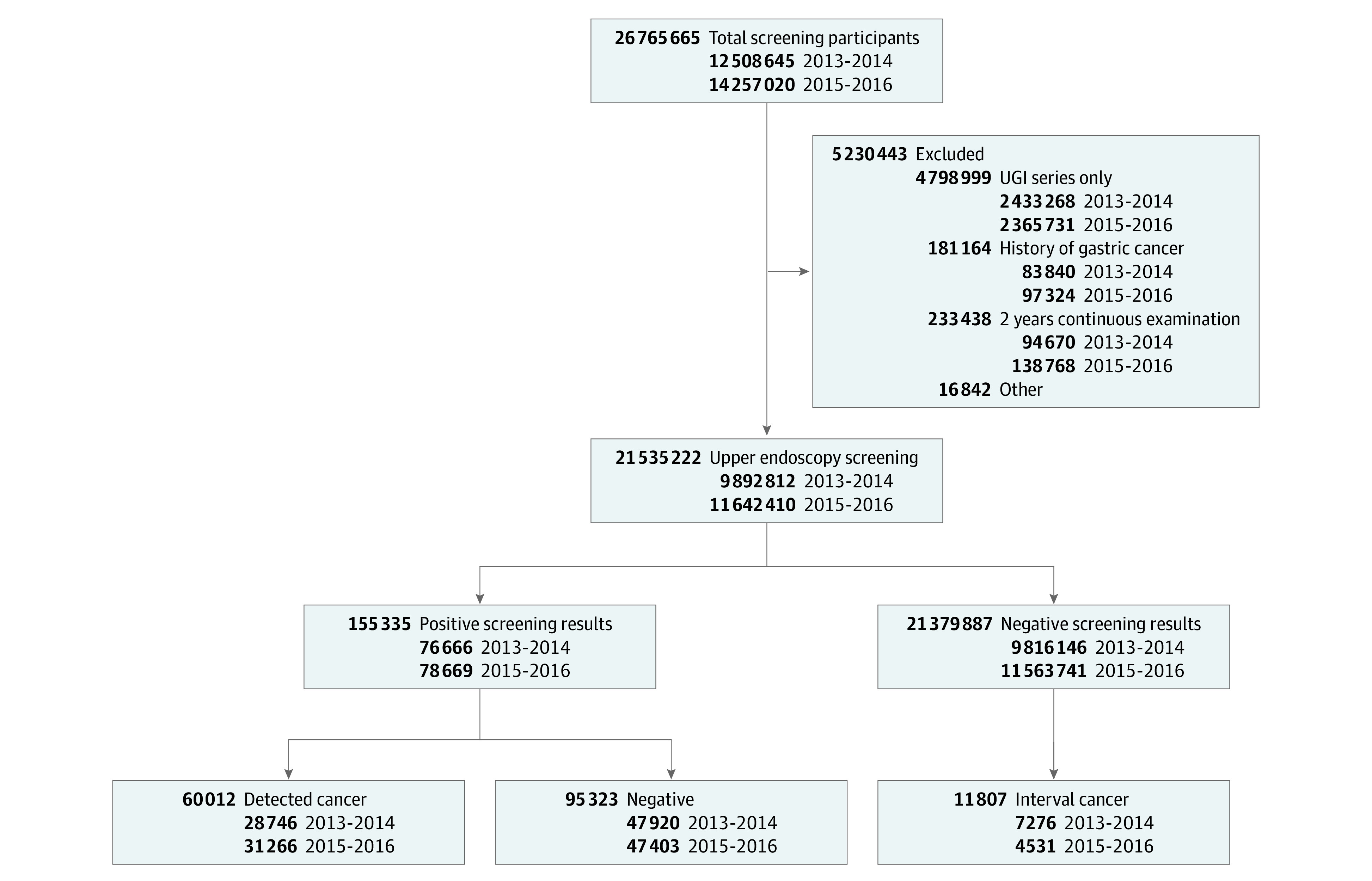
Flow Diagram for Selection of the Screening-Eligible Population Overall, 21 535 222 participants underwent upper endoscopy for gastric cancer screening. A total of 76 666 (0.77%) and 78 669 (0.68%) participants for each cycle screened positive. Of participants who had negative results based on the upper endoscopy, 7276 (0.07%) and 4531 (0.04%) for each respective cycle were diagnosed with gastric cancer within 1 year of endoscopic examination. UGI indicates upper gastrointestinal.

Overall, 26 765 665 participants underwent gastric cancer screening; of these, 12 508 645 individuals (46.73%) underwent screening in 2013-2014 and 14 257 020 individuals (53.27%) underwent screening in 2015-2016. The overall mean (SD) age was 55.61 (10.61) years and 11 761 709 participants (54.62%) were women. A total of 9 892 812 participants for 2013-2014 were selected for inclusion in this analysis, and 11 642 410 were selected for 2015-2016. A total of 766 66 participants (0.77%) and 78 669 participants (0.68%) for each cycle screened positive. Of participants who had negative results based on the upper endoscopy, 7276 participants (0.07%) and 4531 participants (0.04%) for each respective cycle were diagnosed with gastric cancer within 1 year of endoscopic examination. Among those with positive screening results, gastric cancer was diagnosed in 28 746 individuals (37.49%) in 2013-2014 and in 31 266 individuals (39.74%) in 2015-2016 (Figure 1). For the quarterly screening periods, 41.39% individuals (4 094 951) and 42.19% individuals (4 911 629) for each respective cycle underwent endoscopic examinations during the fourth quarter (October-December) (Table 1). The values for the fourth quarter were 2.48 to 2.84 times greater than the number of examinations performed during the first quarter (2013-2014: 1 440 347 [14.56%]; 2015-2016: 1 983 392 [17.03%]). The overall screening performance for gastric cancer detection is reported in eTable 2 in the Supplement.

**Table 1.** Characteristics of Screening-Eligible Population Total, the 2013-2014 and 2015-2016 Korean National Cancer Screening Program Cycles

Characteristics	No. (%)		
	Total	2013-2014 Cycle	2015-2016 Cycle
No. of screenings	21 535 222	9 892 812 (45.94)	11 642 410 (54.06)
History of upper endoscopy			
No	11 518 337 (53.49)	5 303 181 (53.61)	6 215 156 (53.38)
Yes	10 016 885 (46.51)	4 589 631 (46.39)	5 427 254 (46.62)
Sex			
Men	9 773 513 (45.38)	4 438 735 (44.87)	5 334 778 (45.82)
Women	11 761 709 (54.62)	5 454 077 (55.13)	6 307 632 (54.18)
Age, y			
40-49	6 894 681 (32.02)	3 207 074 (32.42)	3 687 607 (31.67)
50-59	6 995 923 (32.49)	3 277 902 (33.13)	3 718 021 (31.94)
60-69	4 907 955 (22.79)	2 184 975 (22.09)	2 722 980 (23.39)
70-79	2 377 279 (11.04)	1 078 948 (10.91)	1 298 331 (11.15)
≥80	359 384 (1.67)	143 913 (1.45)	215 471 (1.85)
Screening period, monthly			
January	796 268 (3.70)	305 062 (3.08)	491 206 (4.22)
February	917 567 (4.26)	400 516 (4.05)	517 051 (4.44)
March	1 709 904 (7.94)	734 769 (7.43)	975 135 (8.38)
April	1 654 273 (7.68)	778 258 (7.87)	876 015 (7.52)
May	1 537 717 (7.14)	739 021 (7.47)	798 696 (6.86)
June	1 333 810 (6.19)	690 117 (6.98)	643 693 (5.53)
July	1 534 662 (7.13)	769 027 (7.77)	765 635 (6.58)
August	1 664 334 (7.73)	773 898 (7.82)	890 436 (7.65)
September	1 380 107 (6.41)	607 193 (6.14)	772 914 (6.64)
October	2 123 625 (9.86)	959 512 (9.70)	1 164 113 (10.00)
November	2 601 103 (12.08)	1 146 148 (11.59)	1 454 955 (12.50)
December	4 281 852 (19.88)	1 989 291 (20.11)	2 292 561 (19.69)
Facility			
General hospital (≥100 beds)	6 461 591 (30.00)	2 991 356 (30.24)	3 470 235 (29.81)
Hospital (30-99 beds)	4 159 574 (19.32)	1 974 047 (19.95)	2 185 527 (18.77)
Clinic (<30 beds)	10 914 057 (50.68)	4 927 409 (49.81)	5 986 648 (51.42)
Screening location 1			
Capital area^a^	10 519 201 (48.85)	4 799 271 (48.51)	5 719 930 (49.13)
Noncapital area	11 016 021 (51.15)	5 093 541 (51.49)	5 922 480 (50.87)
Screening location 2			
Metropolitan area^b^	10 714 042 (49.75)	4 946 492 (50.00)	5 767 550 (49.54)
Nonmetropolitan area	10 821 180 (50.25)	4 946 320 (50.00)	5 874 860 (50.46)
History of gastric disease			
Atrophic gastritis	2 588 607 (12.98)	1 170 883 (12.82)	1 417 724 (13.11)
Ulcer	1 848 897 (9.44)	841 624 (9.39)	1 007 273 (9.48)
Intestinal metaplasia	138 871 (0.74)	54 734 (0.63)	84 137 (0.82)
Gastric polyp	466 239 (2.45)	198 175 (2.28)	268 064 (2.59)
Other	1 898 353 (9.64)	852 132 (9.47)	1 046 221 (9.79)

^a^ The capital area includes Seoul, Gyenggi, and Incheon.

^b^ The metropolitan area includes Seoul, Busan, Incheon, Daegu, Gwangju, Daejeon, and Ulsan.

### Changes in the Number of Participants Screened Monthly

**Figure 2.**
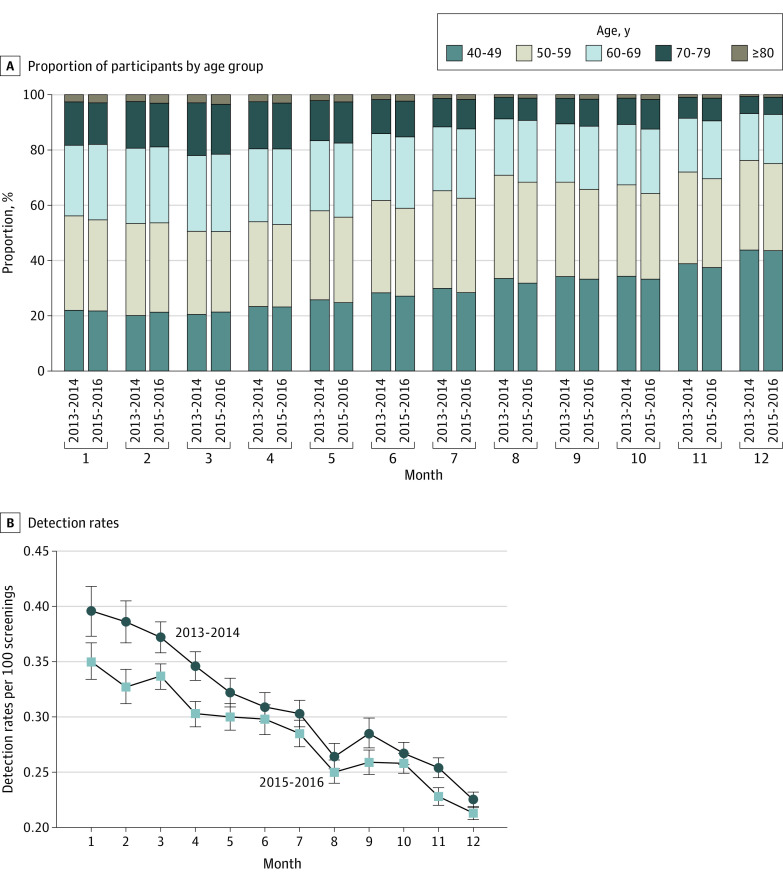
Ratio of Monthly Screenings and Monthly Detection Rates for Gastric Cancer in the Korean National Cancer Screening Program A, Ratio of the monthly screenings based on age grouping for the 2013-2014 cycle and the 2015-2016 cycle. B, Detection rates per 100 screenings decreased toward the end of the year, especially in December. The error bars indicate 95% CIs.

The number of screenings increased from October onward (Table 1). The greatest number of participants were screened in December. For the 2013-2014 cycle, the mean number of participants between January and November was 718 502. In December, the number of participants was 2.77 times greater than the average number screened in the previous 11 months (January-November), and this number increased by 73.56% compared with the number of screenings in November. We observed a similar trend for the number of screenings performed in December for the 2015-2016 cycle (average number of participants between January and November: 849 986; 2.70 times increase in December and 57.57% increase compared with November alone). For the 2013-2014 cycle, the proportions of participants aged 40 to 59 years increased in December (January: 56.17%, December: 76.22%); however, the proportions of participants aged 60 years or older decreased from 43.83% in January to 23.78% in December. These results were similar for the 2015-2016 cycle (age 40-59 years: 54.71% in January vs 75.13% in December; ≥60 years: 45.29% in January vs 24.87% in December) (Figure 2A).

### Monthly Detection Rates for Gastric Cancer

Overall cancer detection rates (2013-2014 and 2014-2015 cycles) showed a tendency to decrease later in the year. The detection rates were the lowest in December (2013-2014: 0.22; 95% CI, 0.22-0.23; 2015-2016: 0.21; 95% CI, 0.21-0.22) when the number of participants screened was the highest; this change was approximately a 40.0% to 45.0% decrease in detection rates compared with the rates in January (Figure 2B). For the 2013-2014 and 2015-2016 cycles, the detection rates decreased with time in the year, especially in December by age group (eFigure in the Supplement). Participants of all ages, with the exception of those in their 60s and 70s, exhibited an approximate 17.83% to 38.46% decrease in detection rates in December compared with January (eTable 3 in the Supplement). The detection rates based on endoscopic history, sex, hospital type, and screening site are reported in eTable 4 in the Supplement.

### Association Between Detection Rates and Calendar Month

Overdispersion was tested, and nonsignificant *P* values (2013-2014: *P* = .45, 2015-2016: *P* = .46) suggested that the negative binomial model was a good fit for the data. Because the proportion of participants based on age group differed from January to December, the age group was added as a covariate in the model along with the month. The age group was a significant factor for both cycles. After adjustment for the age group and taking account of the number of screenings, the estimated coefficient range for the screening month was negative and the detection rate in December was significantly different than in January for both the consequent cycles (2013-2014: −0.05 to −0.18; *P* < .001; and 2015-2016: −0.06 to −0.19; *P* < .001). The detection rates for the older age group were greater than those for the younger age group (Table 2).

**Table 2.** Estimation of Screening Month Effect Size Based on the Negative Binomial Regression^a^

Variable	2013-2014 cycle		2015-2016 cycle	
	β (SE)	*P* value	β (SE)	*P* value
Month		.01		.20
January	0		0	
February	−0.05 (0.04)	.30	−0.09 (0.06)	.14
March	−0.13 (0.04)	<.001	−0.11 (0.06)	.07
April	−0.15 (0.04)	<.001	−0.17 (0.06)	<.001
May	−0.16 (0.04)	<.001	−0.14 (0.06)	.03
June	−0.13 (0.04)	.01	−0.10 (0.06)	.10
July	−0.09 (0.04)	.05	−0.06 (0.06)	.29
August	−0.14 (0.04)	<.001	−0.11 (0.06)	.08
September	−0.11 (0.04)	.03	−0.13 (0.06)	.04
October	−0.18 (0.04)	<.001	−0.16 (0.06)	.01
November	−0.14 (0.05)	<.001	−0.19 (0.06)	<.001
December	−0.18 (0.04)	<.001	−0.16 (0.06)	<.001
Age, y				
40-49	0	<.001	0	<.001
50-59	0.80 (0.03)		0.82 (0.04)	
60-69	1.40 (0.03)		1.38 (0.04)	
70-79	1.98 (0.03)		1.93 (0.04)	
≥80	2.63 (0.04)		2.58 (0.05)	

^a^ The negative binomial regression model was fit with the number of screenings as an offset term. Overdispersion was tested based on scaled Pearson χ^2^ analysis. The covariance matrix was multiplied by a factor of deviance/degree of freedom.

### Factors Associated With Endoscopic Screening Sensitivity

In the univariate analyses, history of endoscopic examination, sex, age group, calendar month, hospital type, metropolitan area, and history of gastric diseases, including atrophic gastritis, ulcer, intestinal metaplasia, gastric polyp, and other gastric diseases, were significantly associated with gastric cancer detection (*P* < .001 for all) (Table 3). In the multivariable logistic regression analysis with selected indicators by the stepwise selection method, these factors remained significant, except hospital type in the 2013-2014 cycle. The overall significance of the associations of the covariates with cancer detection was similar for both cycles. We also found that the screening month was significantly associated with cancer detection (*P* < .001). Compared with January, the odds of cancer detection significantly decreased toward the end of the year for both KNCSP cycles.

**Table 3.** Logistic Regression Analysis of Risk Factors for Gastric Cancer Detection

Variable	2013-2014 Cycle				2015-2016 Cycle			
	Univariate		Multivariable		Univariate		Multivariable	
	OR (95% CI)	*P* value	OR (95% CI)	*P* value	OR (95% CI)	*P* value	OR (95% CI)	*P* value
History of upper endoscopy								
No	1.63 (1.59-1.67)	<.001	1.73 (1.68-1.77)	<.001	1.63 (1.59-1.66)	<.001	1.70 (1.66-1.74)	<.001
Yes	1 [Reference]		1 [Reference]		1 [Reference]		1 [Reference]	
Sex								
Men	2.75 (2.68-2.82)	<.001	2.71 (2.64-2.78)	<.001	2.65 (2.58-2.71)	<.001	2.64 (2.58-2.71)	<.001
Women	1 [Reference]		1 [Reference]		1 [Reference]		1 [Reference]	
Age, y								
40-49	1 [Reference]	<.001	1 [Reference]	<.001	1 [Reference]	<.001	1 [Reference]	<.001
50-59	2.31 (2.20-2.41)		2.39 (2.28-2.50)		2.39 (2.29-2.50)		2.47 (2.36-2.58)	
60-69	4.33 (4.15-4.52)		4.45 (4.26-4.65)		4.27 (4.10-4.46)		4.42 (4.23-4.61)	
70-79	7.71 (7.38-8.06)		7.78 (7.44-8.14)		7.42 (7.11-7.75)		7.56 (7.23-7.90)	
≥80	14.85 (13.98-15.77)		14.47 (13.61-15.37)		14.25 (13.49-15.05)		13.99 (13.23-14.79)	
Month								
January	1 [Reference]	<.001	1 [Reference]	<.001	1 [Reference]	<.001	1 [Reference]	<.001
February	0.98 (0.91-1.07)		0.99 (0.91-1.07)		0.94 (0.87-1.01)		0.95 (0.89-1.03)	
March	0.94 (0.87-1.01)		0.92 (0.86-0.99)		0.96 (0.91-1.03)		0.94 (0.88-1.00)	
April	0.84 (0.78-0.91)		0.88 (0.82-0.95)		0.86 (0.81-0.92)		0.89 (0.83-0.94)	
May	0.80 (0.74-0.86)		0.89 (0.83-0.96)		0.85 (0.79-0.91)		0.91 (0.85-0.97)	
June	0.77 (0.71-0.83)		0.91 (0.84-0.98)		0.83 (0.77-0.89)		0.92 (0.86-0.99)	
July	0.75 (0.69-0.80)		0.93 (0.86-1.00)		0.81 (0.76-0.86)		0.96 (0.90-1.03)	
August	0.65 (0.60-0.70)		0.89 (0.83-0.96)		0.70 (0.66-0.75)		0.92 (0.86-0.98)	
September	0.71 (0.65-0.76)		0.92 (0.85-1.00)		0.72 (0.68-0.77)		0.91 (0.85-0.97)	
October	0.65 (0.61-0.70)		0.84 (0.78-0.91)		0.72 (0.67-0.77)		0.88 (0.83-0.94)	
November	0.63 (0.58-0.67)		0.86 (0.80-0.92)		0.63 (0.60-0.67)		0.84 (0.79-0.89)	
December	0.56 (0.52-0.60)		0.82 (0.76-0.87)		0.59 (0.56-0.63)		0.83 (0.79-0.89)	
Hospital type								
General hospital (≥100 beds)	1 [Reference]	<.001	NA^a^		1 [Reference]	<.001	1 [Reference]	<.001
Hospital (30-99 beds)	1.03 (0.99-1.06)		NA^a^		1.00 (0.97-1.04)		1.02 (0.99-1.06)	
Clinic (<30 beds)	0.97 (0.94-1.00)		NA^a^		0.91 (0.88-0.93)		0.96 (0.94-0.99)	
Metropolitan area^b^								
Yes	0.82 (0.80-0.83)	<.001	0.88 (0.85-0.90)	<.001	0.81 (0.79-0.83)	<.001	0.87 (0.85-0.89)	<.001
No	1 [Reference]		1 [Reference]		1 [Reference]		1 [Reference]	
History of atrophic gastritis								
Yes	0.70 (0.66-0.74)	<.001	0.79 (0.75-0.83)	<.001	0.70 (0.67-0.74)	<.001	0.78 (0.75-0.83)	<.001
No	1 [Reference]		1 [Reference]		1 [Reference]		1 [Reference]	
History of ulcer								
Yes	0.80 (0.75-0.84)	<.001	0.85 (0.80-0.90)	<.001	0.80 (0.76-0.84)	<.001	0.85 (0.80-0.89)	<.001
No	1 [Reference]		1 [Reference]		1 [Reference]		1 [Reference]	
History of intestinal metaplasia								
Yes	1.09 (0.91-1.32)	.35	1.34 (1.12-1.62)	<.001	1.17 (1.01-1.35)	.04	1.40 (1.21-1.62)	.04
No	1 [Reference]		1 [Reference]		1 [Reference]		1 [Reference]	
History of gastric polyp								
Yes	1.35 (1.23-1.48)	<.001	1.27 (1.16-1.39)	<.001	1.26 (1.16-1.36)	<.001	1.16 (1.07-1.26)	<.001
No	1 [Reference]		1 [Reference]		1 [Reference]		1 [Reference]	
History of other gastric diseases								
Yes	0.76 (0.71-0.80)	<.001	0.82 (0.77-0.87)	<.001	0.72 (0.68-0.77)	<.001	0.78 (0.74-0.83)	<.001
No	1 [Reference]		1 [Reference]		1 [Reference]		1 [Reference]	

Abbreviations: NA, not applicable; OR, odds ratio.

^a^ The hospital type was not included in the multivariable analysis of the 2013-2014 cycle after the stepwise selection process.

^b^ The metropolitan area includes Seoul, Busan, Incheon, Daegu, Gwangju, Daejeon, and Ulsan.

## Discussion

This study assessed the association between gastric cancer detection rates and calendar month using data collected from the KNCSP. The findings suggest that there was a greater number of upper endoscopy screening examinations performed during the last quarter of the year, especially in December. Most participants examined in December were aged 40 to 59 years. Gastric cancer detection rates were the lowest in December, although the greatest numbers of individuals were examined in this month. These findings were consistent regardless of age grouping and calendar cycle. We also noted that detection rates were negatively and significantly associated with calendar month. Therefore, the present findings suggest that the rapid increase in the number of screening examinations might increase endoscopists’ workloads and consequently reduce cancer detection rates.

Upper endoscopy is the most effective examination for early diagnosis of gastric cancer.^22,23^ More specifically, endoscopy for screening is associated with lowering gastric cancer–related mortality.^3^ Furthermore, because gastric cancer prevalence increases with age,^24^ endoscopic evaluation as a cancer screening tool is becoming more important. Therefore, the demand for upper endoscopy is rapidly increasing the workload of the limited numbers of endoscopists. Several studies have reported that physician fatigue from being overworked affects their performance.^25,26,27,28,29^ Nevertheless, these studies have limitations, including the small patient numbers, single-center bases, and the limited number of physicians performing endoscopy. Our study used extensive data of approximately 20 million participants from the KNCSP; therefore, the outcomes have important implications.

In the KNCSP, the number of young participants increased sharply toward December. In December, participants aged 40 to 59 years accounted for 76.22% of the included participants in the 2013-2014 cycle and 75.13% of the included participants in the 2015-2016 cycle, which was 8.84 and 6.41 times higher than the frequencies of such individuals in January. Although it is difficult to clearly explain the reason for this finding, we surmise that there may be a psychological factor associated with participants delaying the screening dates as much as possible. In addition, we think that holidays do not have an effect on the monthly variation in screening because the first and the second halves of the year have no major variations in the number of holidays in Korea. Because there was a higher number of participants with a lower gastric cancer prevalence, the decreasing rate of gastric cancer detection toward December may be natural. However, we divided the participants into 5 age groups to determine whether these findings could be explained by an increased number of younger participants. Our findings showed that detection rates decreased toward the end of the year, regardless of age group. Accordingly, we suggest that the decrease in gastric cancer detection rates toward the end of the year could not be attributed to an increased number of younger participants with a relatively lower gastric cancer prevalence.

To reduce the selection bias due to the year of selection, we added the 2 most recent consecutive cycles from the NHIS-NHID; however, the trends were consistent regardless of cycle. Meanwhile, when the overall screening performance was investigated for the 2015-2016 cycle (eTable 2 in the Supplement), the detection rates decreased, sensitivity increased, and interval cancer rates decreased when compared with the 2013-2014 cycle. In Korea, the Ministry of Health and Welfare implemented the endoscopy quality management project of the KNCSP from 2008 to 2014. The 2012-2014 cycle was the second project cycle and, based on the results from the first project cycle, written and on-site evaluations were conducted to improve endoscopy quality management. Since 2016, the third national cancer management project has been in progress. In the KNCSP for gastric cancer, the increase in sensitivity in 2015-2016 can be considered as a positive result owing to the endoscopic quality management policy background. However, despite policies to improve the quality of endoscopic examinations, the detection rates decreased at the end of the year and the proportion of people in the young age group increased in the last quarter of the year for both cycles.

In our study, living in the metropolitan area appeared to be associated with a reduced risk of gastric cancer detection. We assumed that gastric cancer prevalence is lower in this region owing to a high proportion of young individuals. Participants older than 80 years received more endoscopies in the nonmetropolitan area (57.0%) compared with those aged 40 to 49 years (49.0%). If further analysis were possible using cancer information and participants factor, a clearer reason might be found.

### Limitations

This study has several limitations. First, aside from the participants’ gastric history, we did not analyze the association between participant-level factors and gastric cancer detection. Participant factors, including symptoms, may influence the timing of the examination; however, we could not confirm this from the KNCSP’s available data. We could not obtain the information on the participants’ comorbidities; therefore, it could not be included in the logistic regression analysis. Second, this study was based on data from 2013 to 2016—these were the latest data we could access. Although this was a study containing real-world data for more than 20 million individuals with confirmation of cancer occurrence and follow-up records, our findings may not be sufficiently representative. Additional studies assessing the entire cohort of individuals who have undergone subsequent screening may clarify the study outcomes. Third, an objective evaluation of the fatigue experienced by endoscopists was not performed. Fourth, cancer stages and their possible effects were not analyzed in this study owing to data unavailability. Fifth, because this study used screening program data, there may be selection bias.

## Conclusions

We used national population-based data from the KNCSP database to observe a decrease in the gastric cancer detection rates toward the end of the year as the number of examinations rapidly increased. Our findings suggest that policy guidelines are needed to support improvements to screening programs for gastric cancer detection using endoscopy. Such adapted guidelines could contain recommendations on maintaining limits of the number of examinations allowed within a specific period. Further studies should be performed to determine the development index of operator workload or burnout in professionals performing UGI endoscopy to assist in establishing guidelines highlighting the best performance of each operator.

## Notes

Supplement. **eMethods.** Supplemental Methods **eTable 1.** Monthly Number of the Korean National Cancer Screening Program Participating Center **eTable 2.** Overall Screening Performance for Gastric Cancer Between the 2013-2014 and 2015-2016 Korean National Cancer Screening Program Cycles **eTable 3.** Monthly Detection Rates for Gastric Cancer Based on Age Grouping **eTable 4.** Gastric Cancer Detection Rates and 95% Confidence Intervals for Endoscopic Screening Based on a History of Endoscopy, Sex, Age Grouping, Hospital Type, Screening Type, and Month **eFigure.** Monthly Detection Rates for Gastric Cancer Divided Based on Age Grouping in The Korean National Cancer Screening Program **eAppendix 1.** Detailed Statistical Methods for Table 2 **eAppendix 2.** Detailed Statistical Methods for Table 3
