# Supplementary material for: Association of Intensive Endoscopic Screening Burden With Gastric Cancer Detection
Source: JAMA Netw Open. 2021 Jan 7;4(1):e2032542. doi: 10.1001/jamanetworkopen.2020.32542 (PMC7791358; doi:10.1001/jamanetworkopen.2020.32542)
Supplement: Supplement. — eMethods. Supplemental Methods eTable 1. Monthly Number of the Korean National Cancer Screening Program Participating Center eTable 2. Overall Screening Performance for Gastric Cancer Between the 2013-2014 and 2015-2016 Korean National Cancer Screening Program Cycles eTable 3. Monthly Detection Rates for Gastric Cancer Based on Age Grouping eTable 4. Gastric Cancer Detection Rates and 95% Confidence Intervals for Endoscopic Screening Based on a History of Endoscopy, Sex, Age Grouping, Hospital Type, Screening Type, and Month eFigure. Monthly Detection Rates for Gastric Cancer Divided Based on Age Grouping in The Korean National Cancer Screening Program eAppendix 1. Detailed Statistical Methods for Table 2 eAppendix 2. Detailed Statistical Methods for Table 3 [file jamanetwopen-e2032542-s001.pdf]

Supplementary Online Content

Noh CK, Lee E, Lee GH, et al. Association of intensive endoscopic screening burden with gastric cancer detection. *JAMA Netw Open*. 2021;4(1):e2032542. doi:10.1001/jamanetworkopen.2020.32542

eMethods. Supplemental Methods
eTable 1. Monthly Number of the Korean National Cancer Screening Program Participating Center
eTable 2. Overall Screening Performance for Gastric Cancer Between the 2013-2014 and 2015-2016 Korean National Cancer Screening Program Cycles
eTable 3. Monthly Detection Rates for Gastric Cancer Based on Age Grouping
eTable 4. Gastric Cancer Detection Rates and 95% Confidence Intervals for Endoscopic Screening Based on a History of Endoscopy, Sex, Age Grouping, Hospital Type, Screening Type, and Month
eFigure. Monthly Detection Rates for Gastric Cancer Divided Based on Age Grouping in The Korean National Cancer Screening Program
eAppendix 1. Detailed Statistical Methods for Table 2
eAppendix 2. Detailed Statistical Methods for Table 3

This supplementary material has been provided by the authors to give readers additional information about their work.

© 2021 Noh CK et al. *JAMA Network Open.*

eMethods. Supplemental Methods

1. Targeted population with Korean National Cancer Screening Program for six common cancers
Stomach: aged 40 years or older,
Liver: aged 40 years or older with high-risk group of liver cancer,
Colorectum: aged 50 years or older,
Breast: aged 40 years or older,
Uterine cervix: aged 20 years or older,
Lung: aged 54-74 years with a high risk of lung cancer

2. Number of the Korean National Cancer Screening Program participating center for gastric cancer
The average number of institutions per month (data from the National Health Insurance Service)
Year 2013: There were 1,088 institution-level centers (hospitals and general hospitals) and 3,184 clinic-level centers participating in the program.
Year 2014: There were 1,104 institution-level centers (hospitals and general hospitals) and 3,328 clinic-level centers participating in the program.
Year 2015: There were 1,107 institution-level centers (hospitals and general hospitals) and 3,622 clinic-level centers participating in the program.
Year 2016: There were 1,112 institution-level centers (hospitals and general hospitals) and 3,622 clinic-level centers participating in the program.

3. Screening endoscopy protocol in the Korean National Cancer Screening Program
An endoscopic examination involves acquiring images after close observations by an endoscopist, for which imaging of at least eight areas (duodenum, ampulla, antrum, angle, corpus, fundus with retroflexion view, esophagogastric junction, and esophagus) is recommended, along with multiple additional images in areas with abnormal lesions. A tissue biopsy is performed when abnormal findings are detected in the endoscopic examination. If further evaluation or treatment is needed based on the biopsy or endoscopic findings, then the patient is referred to an upper-tier hospital for re-examination.
The endoscopic results were reported according to nine categories: (1) negative, (2) gastritis, (3) possible gastric cancer, (4) early gastric cancer, (5) advanced gastric cancer, (6) gastric ulcer, (7) gastric polyp, (8) subepithelial tumor, and (9) others. As needed, a biopsy was performed, and results were reported according to eight categories: (1) negative, (2) gastritis, (3) inflammation or hyperplastic lesions, (4) low-grade dysplasia, (5) high-grade dysplasia, (6) suspicious gastric cancer, (7) gastric cancer, and (8) others. The cancer screening overall results were reported according to five categories: (1) negative, (2) benign disease, (3) suspicious gastric cancer, (4) gastric cancer, and (5) others.

© 2021 Noh CK et al. *JAMA Network Open.*

eTable 1. Monthly Number of the Korean National Cancer Screening Program Participating Center (Data from the National Health Insurance Service)

Year	Center	Jan	Feb	Mar	Apr	May	Jun	Jul	Aug	Sep	Oct	Nov	Dec
2013	General hospital	306	305	307	308	305	*[a]	*	*	*	*	307	307
	Hospital	775	771	782	788	791	*	*	*	*	*	779	782
	Clinics	3,116	3,132	3,148	3,164	3,200	*	*	*	*	*	3,262	3,267
	Other	8	8	8	8	8	*	*	*	*	*	8	8
	Total	4,205	4,216	4,245	4,268	4,304	*	*	*	*	*	4,356	4,364
2014	General hospital	307	308	312	311	311	313	315	316	316	315	315	317
	Hospital	779	781	783	786	794	795	795	792	791	799	798	793
	Clinics	3,260	3,264	3,276	3,305	3,307	3,317	3,323	3,343	3,362	3,385	3,395	3,400
	Other	8	8	8	8	8	8	8	8	8	8	8	8
	Total	4,354	4,361	4,379	4,410	4,420	4,433	4,441	4,459	4,477	4,507	4,516	4,518
2015	General hospital	318	318	322	322	323	322	322	323	322	323	326	325
	Hospital	787	791	798	803	794	789	778	778	775	774	772	782
	Clinics	3,400	3,398	3,425	3,441	3,450	3,460	3,481	3,489	3,516	3,525	3,537	3,548
	Other	8	8	8	8	8	8	8	8	8	8	8	8
	Total	4,513	4,515	4,553	4,574	4,575	4,579	4,589	4,598	4,621	4,630	4,643	4,663
2016	General hospital	326	326	325	324	324	326	327	*	327	327	329	328
	Hospital	777	772	782	789	794	791	791	*	788	790	786	784
	Clinics	3,543	3,554	3,583	3,598	3,611	3,616	3,632	*	3,652	3,672	3,686	3,690
	Other	8	8	8	8	8	8	8	*	7	7	7	7
	Total	4,654	4,660	4,698	4,719	4,737	4,741	4,758	*	4,774	4,796	4,808	4,809

[a] Data did not exist.

© 2021 Noh, C-K, et al. *JAMA Network Open.*

eTable 2. Overall Screening Performance for Gastric Cancer Between The 2013-2014 and 2015-2016 Korean National Cancer Screening Program Cycles[a]

**2013-2014 cycle**	
Sensitivity	0.798 (0.794-0.802)
Specificity	0.995 (0.995-0.995)
Positive predictive value	0.375 (0.371-0.378)
Detection rates (per 100)	0.291 (0.171-0.287)
Interval cancer rates (per negative 1,000)	0.741 (0.724-0.758)
Positive rates (per 1,000)	7.750 (7.695-7.804)
**2015-2016 cycle**	
Sensitivity	0.873 (0.870-0.877)
Specificity	0.996 (0.996-0.996)
Positive predictive value	0.397 (0.394-0.401)
Detection rates (per 100)	0.269 (0.266-0.272)
Interval cancer rates (per negative 1,000)	0.392 (0.380-0.403)
Positive rates (per 1,000)	6.757 (6.710-6.804)

[a] Presented with 95% confidence interval.

© 2021 Noh, C-K, et al. *JAMA Network Open.*

eTable 3. Monthly Detection Rates for Gastric Cancer Based on Age Grouping

Age	2013-2014 cycle											
	Jan	Feb	Mar	Apr	May	Jun	Jul	Aug	Sep	Oct	Nov	Dec
40-49	0.13	0.13	0.12	0.11	0.11	0.11	0.10	0.10	0.09	0.09	0.09	0.08
50-59	0.27	0.25	0.23	0.22	0.22	0.22	0.23	0.21	0.22	0.21	0.21	0.21
60-69	0.43	0.41	0.40	0.38	0.37	0.38	0.41	0.40	0.42	0.38	0.43	0.42
70-79	0.80	0.77	0.69	0.70	0.69	0.71	0.75	0.73	0.79	0.69	0.72	0.69
≥80	1.57	1.47	1.30	1.30	1.29	1.45	1.68	1.48	1.30	1.34	1.40	1.29
**Age**	**2015-2016 cycle**											
	**Jan**	**Feb**	**Mar**	**Apr**	**May**	**Jun**	**Jul**	**Aug**	**Sep**	**Oct**	**Nov**	**Dec**
40-49	0.12	0.11	0.11	0.10	0.11	0.10	0.10	0.08	0.08	0.08	0.07	0.08
50-59	0.25	0.23	0.23	0.20	0.20	0.22	0.21	0.18	0.21	0.20	0.19	0.19
60-69	0.39	0.36	0.35	0.33	0.33	0.37	0.37	0.37	0.36	0.37	0.36	0.39
70-79	0.62	0.61	0.62	0.58	0.63	0.62	0.64	0.69	0.64	0.60	0.64	0.62
≥80	1.42	1.13	1.04	1.12	1.13	1.11	1.50	1.45	1.37	1.24	1.08	1.15

© 2021 Noh, C-K, et al. *JAMA Network Open.*

eTable 4. Gastric Cancer Detection Rates and 95% Confidence Intervals for Endoscopic Screening Based on a History of Endoscopy, Sex, Age Grouping, Hospital Type, Screening Type, and Month

Variables	2013-2014 cycle		2015-2016 cycle	
	Detection rates	95% CI	Detection rates	95% CI
Overall	0.29	(0.17, 0.29)	0.27	(0.27, 0.27)
Gender				
Male	0.45	(0.44, 0.45)	0.40	(0.40, 0.41)
Female	0.16	(0.16, 0.17)	0.15	(0.15, 0.16)
History of upper endoscopy				
No history	0.35	(0.35, 0.36)	0.33	(0.32, 0.33)
History	0.22	(0.21, 0.22)	0.20	(0.20, 0.21)
Age group, years				
40-49	0.10	(0.09, 0.10)	0.09	(0.08, 0.09)
50-59	0.22	(0.21, 0.22)	0.20	(0.20, 0.21)
60-69	0.40	(0.39, 0.41)	0.36	(0.36, 0.37)
70-79	0.72	(0.70, 0.73)	0.62	(0.61, 0.64)
≥ 80	1.38	(1.32, 1.44)	1.19	(1.15, 1.24)
Hospital type				
General hospital	0.29	(0.29, 0.30)	0.28	(0.28, 0.29)
Hospital	0.30	(0.29, 0.31)	0.28	(0.28, 0.29)
Clinics	0.28	(0.28, 0.29)	0.26	(0.25, 0.26)

© 2021 Noh, C-K, et al. *JAMA Network Open.*

Public health center	0.28	(0.28, 0.29)	0.26	(0.25, 0.26)
Screening site 1				
Capital area[a]	0.28	(0.27, 0.28)	0.26	(0.25, 0.26)
Other	0.30	(0.30, 0.31)	0.28	(0.28, 0.28)
Screening site 2				
Metropolitan area[b]	0.26	(0.26, 0.27)	0.24	(0.24, 0.24)
Other	0.32	(0.31, 0.32)	0.30	(0.29, 0.30)
Month				
January	0.40	(0.37, 0.42)	0.35	(0.33, 0.37)
February	0.39	(0.37, 0.41)	0.33	(0.31, 0.34)
March	0.37	(0.36, 0.39)	0.34	(0.33, 0.35)
April	0.35	(0.33, 0.36)	0.30	(0.29, 0.31)
May	0.32	(0.31, 0.34)	0.30	(0.29, 0.31)
June	0.31	(0.30, 0.32)	0.30	(0.28, 0.31)
July	0.30	(0.29, 0.32)	0.29	(0.27, 0.30)
August	0.26	(0.25, 0.28)	0.25	(0.24, 0.26)
September	0.29	(0.27, 0.30)	0.26	(0.25, 0.27)
October	0.27	(0.26, 0.28)	0.26	(0.25, 0.27)
November	0.25	(0.25, 0.26)	0.23	(0.22, 0.24)
December	0.22	(0.22, 0.23)	0.21	(0.21, 0.22)

Abbreviations: CI, confidence interval.
[a] The Capital area includes Seoul, Gyenggi, Incheon.
[b] The Metropolitan area includes Seoul, Busan, Incheon, Daegu, Gwangju, Daejeon, Ulsan.

eFigure. Monthly Detection Rates for Gastric Cancer Divided Based on Age Grouping in The Korean National Cancer Screening Program (40–49; 50–59; 60–69; 70–79; ≥80 years)

Monthly Detection Rates for Gastric Cancer Divided Based on The (A) 2013 – 2014 Cycle and (B) 2015 –2016 Cycle.

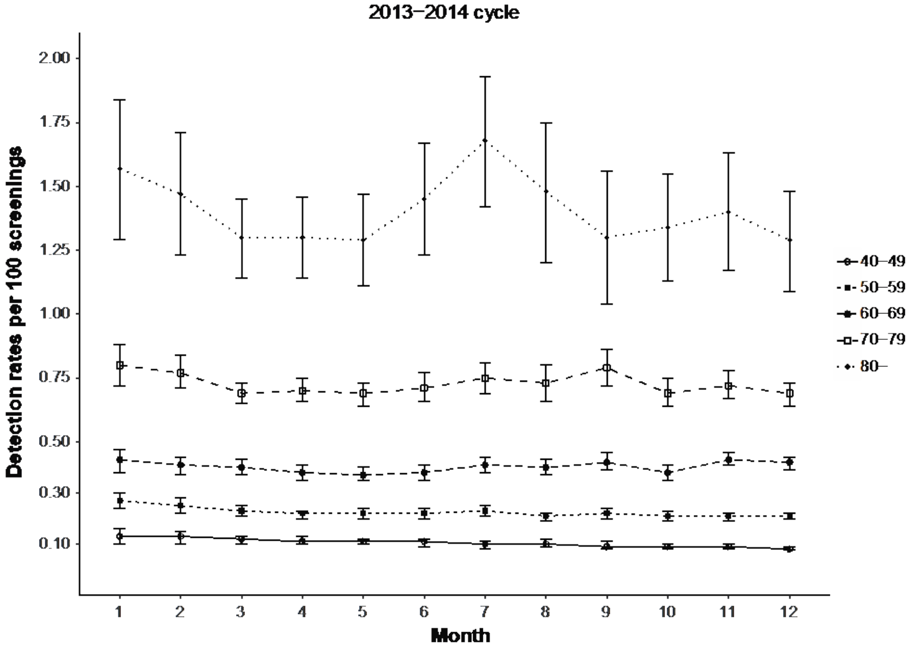

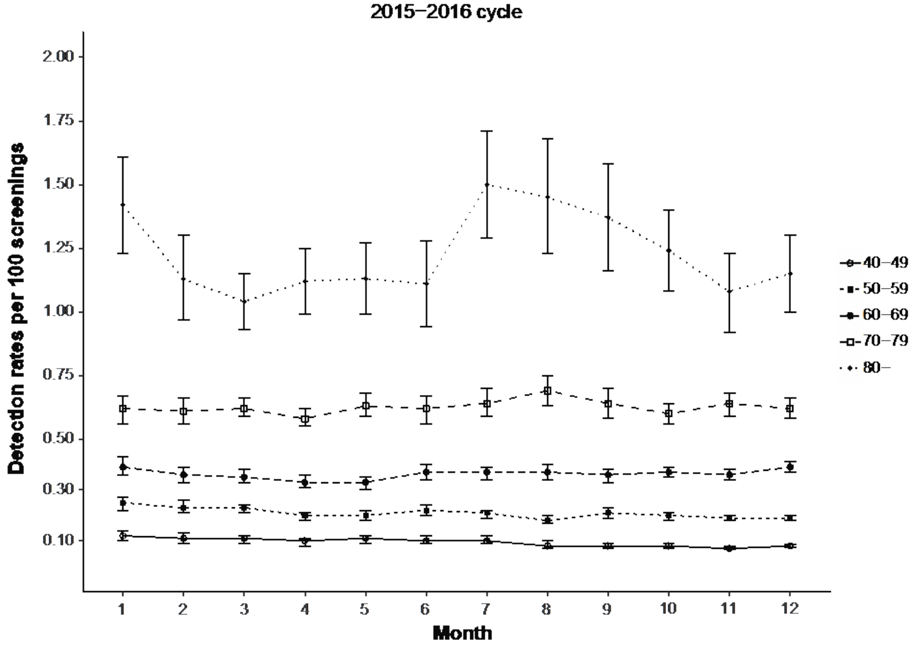

© 2021 Noh, C-K, et al. *JAMA Network Open.*

eAppendix 1. Detailed Statistical Methods for Table 2

Modeling proportion data

Purpose of the analysis: to assess monthly detection rates using a negative binomial regression analysis.

• Sample size: N= 9,892,812 for 2013-2014 cycle, 11,642,410 for 2015-2016 cycle

• Variables used in the analysis: screening month and age group

Characteristics	2013-2014 cycle	2015-2016 cycle
Age (year), No. (%)		
40-49	3,207,074 (32.42)	3,687,607 (31.67)
50-59	3,277,902 (33.13)	3,718,021 (31.94)
60-69	2,184,975 (22.09)	2,722,980 (23.39)
70-79	1,078,948 (10.91)	1,298,331 (11.15)
≥80	143,913 (1.45)	215,471 (1.85)
Screening period, monthly, No. (%)		
January	305,062 (3.08)	491,206 (4.22)
February	400,516 (4.05)	517,051 (4.44)
March	734,769 (7.43)	975,135 (8.38)
April	778,258 (7.87)	876,015 (7.52)
May	739,021 (7.47)	798,696 (6.86)
June	690,117 (6.98)	643,693 (5.53)
July	769,027 (7.77)	765,635 (6.58)
August	773,898 (7.82)	890,436 (7.65)
September	607,193 (6.14)	772,914 (6.64)
October	959,512 (9.7)	1,164,113 (10)
November	1,146,148 (11.59)	1,454,955 (12.5)
December	1,989,291 (20.11)	2,292,561 (19.69)

© 2021 Noh, C-K, et al. *JAMA Network Open.*

	2013-2014 cycle			2015-2016 cycle		
**Month**	**Detected cancer cases**	**Detection rates (per 100)**	**Total N**	**Detected cancer cases**	**Detection rates (per 100)**	**Total N**
1	1,207	0.396	305,062	1,720	0.350	491,206
2	1,546	0.386	400,516	1,693	0.327	517,051
3	2,734	0.372	734,769	3,283	0.337	975,135
4	2,690	0.346	778,258	2,653	0.303	876,015
5	2,377	0.322	739,021	2,396	0.300	798,696
6	2,133	0.309	690,117	1,915	0.298	643,693
7	2,330	0.303	769,027	2,183	0.285	765,635
8	2,045	0.264	773,898	2,228	0.250	890,436
9	1,733	0.285	607,193	2,001	0.259	772,914
10	2,563	0.267	959,512	3,002	0.258	1,164,113
11	2,913	0.254	1,146,148	3,317	0.228	1,454,955
12	4,475	0.225	1,989,291	4,875	0.213	2,292,561
All	28,746	0.291	9,892,812	31,266	0.269	11,642,410

• All analysis by SAS procedures handle missing values by omitting the missing values.
• Regression equation:
Let
d = the number of detected cancers per month
N = the total number of endoscopic screenings per month
Then, the rate of occurrence, i.e. monthly detected cancer = d/N with the expected value of

$$\mathrm{E}\left(\frac{d}{N}\right) = \frac{1}{N}E(d) = \frac{\mu}{N}$$

The Negative binomial regression model with log link for the expected rate of occurrence is

$$\log\left(\frac{d}{N}\right) = \beta_0 + \sum_{i=1}^{17} \beta_i x_i$$

© 2021 Noh, C-K, et al. *JAMA Network Open.*

$$\log(d) - \log(\mathrm{N}) = \sum_{i=1}^{17} \beta_i x_i$$

$$\log(d) = \sum_{i=1}^{17} \beta_i x_i + \log(N)$$

where

$\beta_{1,2,3,4,5,6,7,8,9,10,11} =$ coefficient for calendar month from February to December, respectively.

$\beta_{12,13,14,15}$ = coefficient for age group; 50-59, 60-69, 70-79, 80-

$x_{1,2,3,4,5,6,7,8,9,10,11} =$ 1 for screening month, Feb, …, Dec., respectively; else 0

$x_{12,13,14,15}$ =1 for age group, 50-59, 60-69, 70-70, 80- , respectively; else 0

The term log(N) is an adjustment term, which is called offset.

• Goodness-of-fit: The goodness-of-fit was test by scaled Pearson's χ^2. (*P*-value = 0.44728 for 2013-2014 cycle, 0.46407 for 2015-2016 cycle)

Criteria for assessing Goodness-of-fit

**Criterion**	**2013-2014 cycle**			**2015-2016 cycle**		
	**DF**	**Values**	**Values/DF**	**DF**	**Values**	**Values/DF**
Deviance	44	59.6328	1.3553	44	66.9843	1.5224
Scaled Deviance	44	59.6328	1.3553	44	66.9843	1.5224
Pearson Chi-Square	44	60.4178	1.3731	44	67.2567	1.5286
Scaled Pearson X2	44	44.5792	1.0132	44	44.1790	1.0041
Log Likelihood		154198.5			169872.8	
Full Log Likelihood		-272.654			-297.802	
AIC (smaller is better)		579.308			629.604	
AICC (smaller is better)		593.8795			644.1755	
BIC (smaller is better)		614.9119			665.2079	

• The detected cancers are affected by the number of screenings. When this occurs, we may want to take into account the "denominator" and form a rate, *i.e.* monthly detection rate. To detect overall trends in monthly detection rates as a response variable, we could apply either Poisson regression analysis or negative binomial regression analysis for such a rate data. We applied a Poisson regression model first. But, overdispersion was observed. The model was better fit with negative binomial regression analysis.

Additionally, we added an interaction term between month and age group, but goodness-of-fit was not assessed.

Criteria for assessing Goodness-of-fit for model with interaction term

**Criterion**	**2013-2014 cycle**			**2015-2016 cycle**		
	**DF**	**Values**	**Values/DF**	**DF**	**Values**	**Values/DF**
Deviance	0	0.0000	.	0	0.0000	.
Scaled Deviance	0	0.0000	.	0	0.0000	.
Pearson Chi-Square	.	0.0000	.	.	0.0000	.
Scaled Pearson X2	.	0.0000	.	.	0.0000	.
Log Likelihood		154054.1492			169746.9912	
Full Log Likelihood		-4164.9636			-423.6194	
AIC (smaller is better)		953.9272			967.2388	
AICC (smaller is better)		.			.	
BIC (smaller is better)		1079.5879			1092.8995	

• Analysis of maximum likelihood parameter estimates

< 2013-2014 cycle >

**Parameter**	**DF**	**Estimate**	**Standard Error**	**Wald 95% Confidence Limits**		**Wald Chi-Square**	**Pr > ChiSq**
Intercept	1	-6.7864	0.0451	-6.8748	-6.6979	22634.5	<.0001
Month, Jan	0	0	0	0	0		
Month, Feb	1	0.0521	0.0504	-0.1509	0.0467	1.07	0.3017
Month, Mar	1	-0.1322	0.0464	-0.2232	-0.0411	8.10	0.0044
Month, Apr	1	-0.1523	0.0465	-0.2433	-0.0612	10.74	0.0011
Month, May	1	-0.1581	0.0471	-0.2505	-0.0658	11.26	0.0008
Month, Jun	1	-0.1342	0.0478	-0.2279	-0.0405	7.87	0.0050
Month, Jul	1	-0.0946	0.0473	-0.1873	-0.0020	4.00	0.0454
Month, Aug	1	-0.1370	0.0482	-0.2315	-0.0425	8.08	0.0045
Month, Sep	1	-0.1068	0.0495	-0.2038	-0.0098	4.66	0.0309
Month, Oct	1	-0.1784	0.0467	-0.2700	-0.0868	14.57	0.0001
Month, Nov	1	-0.1447	0.0462	-0.2352	-0.0541	9.81	0.0017
Month, Dec	1	-0.1841	0.0446	-0.2715	-0.0968	17.07	<.0001
Age group, 40-49	0	0.0000	0.0000	0.0000	0.0000	.	.
Age group, 50-59	1	0.8008	0.0300	0.7421	0.8596	714.76	<.0001
Age group, 60-69	1	1.4011	0.0302	1.3419	1.4603	2151.03	<.0001

© 2021 Noh, C-K, et al. *JAMA Network Open.*

Age group, 70-79	1	1.9819	0.0300	1.9230	2.0407	4355.51	<.0001
Age group, 80-	1	2.6348	0.0377	2.5609	2.7087	4886.25	<.0001
Dispersion	1	0.0008	0.0006	0.0002	0.0035		

© 2021 Noh, C-K, et al. *JAMA Network Open.*

< 2015-2016 cycle >

Parameter	DF	Estimate	Standard Error	Wald 95% Confidence Limits		Wald Chi-Square	Pr > ChiSq
Intercept	1	-6.8797	0.0548	-6.9871	-6.7723	15753.0	<.0001
Month, Jan	0						
Month, Feb	1	-0.0939	0.0635	-0.2184	0.0306	2.19	0.1393
Month, Mar	1	-0.1098	0.0598	-0.2269	0.0073	3.38	0.0662
Month, Apr	1	-0.1734	0.0607	-0.2924	-0.0544	8.16	0.0043
Month, May	1	-0.1361	0.0612	-0.2560	-0.0161	4.94	0.0262
Month, Jun	1	-0.1030	0.0626	-0.2257	0.0197	2.71	0.0998
Month, Jul	1	-0.0647	0.0618	-0.1857	0.0563	1.10	0.2947
Month, Aug	1	-0.1095	0.0618	-0.2305	0.0116	3.14	0.0763
Month, Sep	1	-0.1280	0.0623	-0.2500	-0.0060	4.23	0.0398
Month, Oct	1	-0.1567	0.0601	-0.2745	-0.0389	6.80	0.0091
Month, Nov	1	-0.1940	0.0599	-0.3113	-0.0766	10.49	0.0012
Month, Dec	1	-0.1558	0.0585	-0.2705	-0.0412	7.09	0.0077
Age group, 40-49	0	0.0000	0.0000	0.0000	0.0000	.	.
Age group, 50-59	1	0.8195	0.0404	0.7404	0.8987	411.64	<.0001
Age group, 60-69	1	1.3781	0.0400	1.2998	1.4565	1189.57	<.0001

© 2021 Noh, C-K, et al. *JAMA Network Open.*

Age group, 70-79	1	1.9270	0.0403	1.8480	2.0060	2285.65	<.0001
Age group, 80-	1	2.5835	0.0450	2.4954	2.6717	3299.79	<.0001
Dispersion	1	0.0032	0.0014	0.0014	0.0074		

• Analyses were performed using SAS version 9.4 (SAS Institute Inc., Cary, NC, USA).
The code for this model is:

© 2021 Noh, C-K, et al. *JAMA Network Open.*

```
*---------------- Table 2 --------------;

*---------------------------------------;
/* negative binomial regression model */
*---------------------------------------;
data dr_ag;
   infile datalines dlm='#';
   input month n age_gr $ year dc;
   ln = log(n);
 datalines;
1       #67071        #40-49 #1314  #86
2       #80412        #40-49 #1314  #103
3       #150538       #40-49 #1314  #175
4       #181814       #40-49 #1314  #203
5       #190053       #40-49 #1314  #209
6       #195309       #40-49 #1314  #209
7       #229877       #40-49 #1314  #220
8       #259123       #40-49 #1314  #268
9       #207700       #40-49 #1314  #194
10      #328933       #40-49 #1314  #308
11      #444740       #40-49 #1314  #393
12      #871504       #40-49 #1314  #722
1       #104289       #50-59 #1314  #283
2       #133444       #50-59 #1314  #334
3       #220717       #50-59 #1314  #509
4       #238547       #50-59 #1314  #516
5       #238507       #50-59 #1314  #531
6       #230937       #50-59 #1314  #508
7       #271664       #50-59 #1314  #625
8       #289382       #50-59 #1314  #598
9       #207229       #50-59 #1314  #455
10      #317321       #50-59 #1314  #667
11      #381056       #50-59 #1314  #784
12      #644809       #50-59 #1314  #1348
1       #77878        #60-69 #1314  #333
2       #109335       #60-69 #1314  #444
3       #201946       #60-69 #1314  #805
4       #205857       #60-69 #1314  #784
5       #187815       #60-69 #1314  #704
6       #166718       #60-69 #1314  #638
7       #177903       #60-69 #1314  #725
8       #157864       #60-69 #1314  #632
9       #128334       #60-69 #1314  #542
10      #209822       #60-69 #1314  #798
11      #222921       #60-69 #1314  #964
12      #338582       #60-69 #1314  #1409
1       #48104        #70-79 #1314  #384
2       #67537        #70-79 #1314  #521
3       #140124       #70-79 #1314  #967
4       #132493       #70-79 #1314  #933
5       #107380       #70-79 #1314  #736
6       #85577        #70-79 #1314  #610
7       #79677        #70-79 #1314  #594
8       #60072        #70-79 #1314  #437
9       #56569        #70-79 #1314  #446
10      #92066        #70-79 #1314  #638
```

© 2021 Noh, C-K, et al. *JAMA Network Open.*

11	#87318		#70-79	#1314	#630
12	#122031		#70-79	#1314	#837
1	#7720	#80-	#1314	#121	
2	#9788	#80-	#1314	#144	
3	#21444	#80-	#1314	#278	
4	#19547	#80-	#1314	#254	
5	#15266	#80-	#1314	#197	
6	#11576	#80-	#1314	#168	
7	#9906	#80-	#1314	#166	
8	#7457	#80-	#1314	#110	
9	#7361	#80-	#1314	#96	
10	#11370	#80-	#1314	#152	
11	#10113	#80-	#1314	#142	
12	#12365	#80-	#1314	#159	
1	#106868		#40-49	#1516	#132
2	#110217		#40-49	#1516	#120
3	#208026		#40-49	#1516	#224
4	#203302		#40-49	#1516	#196
5	#197176		#40-49	#1516	#209
6	#174237		#40-49	#1516	#177
7	#217029		#40-49	#1516	#222
8	#283429		#40-49	#1516	#239
9	#257193		#40-49	#1516	#195
10	#387388		#40-49	#1516	#303
11	#544541		#40-49	#1516	#397
12	#998201		#40-49	#1516	#793
1	#161871		#50-59	#1516	#401
2	#167031		#50-59	#1516	#388
3	#283734		#50-59	#1516	#643
4	#260968		#50-59	#1516	#516
5	#247735		#50-59	#1516	#502
6	#205243		#50-59	#1516	#446
7	#261665		#50-59	#1516	#542
8	#325202		#50-59	#1516	#597
9	#251174		#50-59	#1516	#525
10	#360845		#50-59	#1516	#710
11	#468324		#50-59	#1516	#886
12	#724229		#50-59	#1516	#1395
1	#134484		#60-69	#1516	#529
2	#142341		#60-69	#1516	#509
3	#273592		#60-69	#1516	#968
4	#240100		#60-69	#1516	#798
5	#214125		#60-69	#1516	#699
6	#166192		#60-69	#1516	#617
7	#192590		#60-69	#1516	#703
8	#199318		#60-69	#1516	#740
9	#176395		#60-69	#1516	#627
10	#271794		#60-69	#1516	#1002
11	#304537		#60-69	#1516	#1085
12	#407512		#60-69	#1516	#1573
1	#73616		#70-79	#1516	#454
2	#81732		#70-79	#1516	#498
3	#176330		#70-79	#1516	#1099
4	#145434		#70-79	#1516	#850
5	#119132		#70-79	#1516	#754
6	#83514		#70-79	#1516	#514
7	#81628		#70-79	#1516	#525

© 2021 Noh, C-K, et al. *JAMA Network Open.*

```
8       #71968        #70-79 #1516  #499
9       #75975        #70-79 #1516  #487
10      #125379       #70-79 #1516  #755
11      #120658       #70-79 #1516  #767
12      #142965       #70-79 #1516  #888
1       #14367 #80-   #1516  #204
2       #15730 #80-   #1516  #178
3       #33453 #80-   #1516  #349
4       #26211 #80-   #1516  #293
5       #20528 #80-   #1516  #232
6       #14507 #80-   #1516  #161
7       #12723 #80-   #1516  #191
8       #10519 #80-   #1516  #153
9       #12177 #80-   #1516  #167
10      #18707 #80-   #1516  #232
11      #16895 #80-   #1516  #182
12      #19654 #80-   #1516  #226
;
run;

%macro rate_data(varlist=  , yr = , dist = );
proc genmod data=dr_ag(where=(year=&yr.));
  class age_gr month / ref=first;
  model dc = &varlist. / type3 dist=&dist. link=log offset= ln dscale ;
run;
%mend;
%rate_data(varlist = month, yr=1314, dist=poi)
%rate_data(varlist = month, yr=1516, dist=poi)
%rate_data(varlist = month, yr=1314, dist=nb)
%rate_data(varlist = month, yr=1516, dist=nb)

%rate_data(varlist = month age_gr, yr=1314, dist=poi)
%rate_data(varlist = month age_gr, yr=1516, dist=poi)
%rate_data(varlist = month age_gr, yr=1314, dist=nb)
%rate_data(varlist = month age_gr, yr=1516, dist=nb)

%rate_data(varlist = month age_gr month*age_gr, yr=1314, dist=poi)
%rate_data(varlist = month age_gr month*age_gr, yr=1516, dist=poi)
%rate_data(varlist = month age_gr month*age_gr, yr=1314, dist=nb)
%rate_data(varlist = month age_gr month*age_gr, yr=1516, dist=nb)
```

© 2021 Noh, C-K, et al. *JAMA Network Open.*

eAppendix 2. Detailed Statistical Methods for Table 3

• Purpose of the analysis: to evaluate the risk factors for cancer detection using a logistic regression analysis.

• Sample size: N= 9,892,812 for 2013-2014 cycle, 11,642,410 for 2015-2016 cycle

• Variables used in the analysis: history of upper endoscopy, sex, age groups, screening month, hospital type, metropolitan area, history of gastric disease; including atrophic gastritis, ulcer, intestinal metaplasia, gastric polyp, other

**Characteristics**	**2013-2014 cycle**	**2015-2016 cycle**
History of upper endoscopy, No. (%)		
No	5,303,181 (53.61)	6,215,156 (53.38)
Yes	4,589,631 (46.39)	5,427,254 (46.62)
Sex, No. (%)		
Male	4,438,735 (44.87)	5,334,778 (45.82)
Female	5,454,077 (55.13)	6,307,632 (54.18)
Age (year), No. (%)		
40-49	3,207,074 (32.42)	3,687,607 (31.67)
50-59	3,277,902 (33.13)	3,718,021 (31.94)
60-69	2,184,975 (22.09)	2,722,980 (23.39)
70-79	1,078,948 (10.91)	1,298,331 (11.15)
≥80	143,913 (1.45)	215,471 (1.85)
Screening period, monthly, No. (%)		
January	305,062 (3.08)	491,206 (4.22)
February	400,516 (4.05)	517,051 (4.44)
March	734,769 (7.43)	975,135 (8.38)
April	778,258 (7.87)	876,015 (7.52)
May	739,021 (7.47)	798,696 (6.86)
June	690,117 (6.98)	643,693 (5.53)

© 2021 Noh, C-K, et al. *JAMA Network Open.*

July	769,027 (7.77)	765,635 (6.58)
August	773,898 (7.82)	890,436 (7.65)
September	607,193 (6.14)	772,914 (6.64)
October	959,512 (9.7)	1,164,113 (10)
November	1,146,148 (11.59)	1,454,955 (12.5)
December	1,989,291 (20.11)	2,292,561 (19.69)
Hospital type, No. (%)		
General hospital	2,991,356 (30.24)	3,470,235 (29.81)
Hospital	1,974,047 (19.95)	2,185,527 (18.77)
Clinics	4,927,409 (49.81)	5,986,648 (51.42)
Screening location, No. (%)		
Metropolitan area[c]	4,946,492 (50.00)	5,767,550 (49.54)
Non-metropolitan area	4,946,320 (50.00)	5,874,860 (50.46)
History of gastric disease		
Atrophic gastritis, No. (%)	1,170,883(12.82)	1,417,724(13.11)
Ulcer, No. (%)	841,624(9.39)	1,007,273(9.48)
Intestinal metaplasia, No. (%)	54,734(0.63)	84,137(0.82)
Gastric polyp, No. (%)	198,175(2.28)	268,064(2.59)
Other, No. (%)	852,132(9.47)	1,046,221(9.79)

• Assumptions of the analysis: the assumptions of appropriate outcome structure, observation independence, the absence of multicollinearity, linearity of independent variables and log odds, and large sample size were met.
• All analysis by SAS procedures handle missing values by omitting the missing values.
• Regression equation:

$$\text{logit} = log\frac{P(Y = detected\ cancer)}{1 + P(Y = detected\ cancer)} = \beta_0 + \sum_{i=1}^{25} \beta_i X_i + \varepsilon$$

© 2021 Noh, C-K, et al. *JAMA Network Open.*

where
$\beta_0 =$ Intercept for logit for those having no history of endoscopy, female, aged 40-49, screened at non-metropolitan area and general hospital, in January, none of history of gastric diseases.
$\beta_1 =$ Incremental effect for having history of endoscopy
$\beta_2 =$ Incremental effect for male
$\beta_{3,4,5,6} =$ Incremental effect for age group, 50-59, 60-69, 70-70, 80- , respectively
$\beta_7 =$ Incremental effect for metropolitan area
$\beta_{8,9,10,11,12,13,14,15,16,17,18} =$ Incremental effect for screening month, Jan, Feb, …, Dec., respectively
$\beta_{19,20} =$ Incremental effect for screening hospitals and clinics, respectively
$\beta_{21} =$ Incremental effect for having history of atrophic gastritis
$\beta_{22} =$ Incremental effect for having history of ulcer
$\beta_{23} =$ Incremental effect for having history of intestinal metaplasia
$\beta_{24} =$ Incremental effect for having history of gastric polyp
$\beta_{25} =$ Incremental effect for having history of other gastric diseases
$x_1 =$ 1 for having history of endoscopy, else 0
$x_2 =$ 1 for male, else 0
$x_{3,4,5,6} =$ 1 for age group, 50-59, 60-69, 70-70, 80- , respectively; else 0
$x_7 =$ 1 for metropolitan area, else 0
$x_{8,9,10,11,12,13,14,15,16,17,18} =$ 1 for screening month, Jan, Feb, …, Dec., respectively; else 0
$x_{19,20} =$ 1 for screening hospitals and clinics, respectively; else 0
$x_{21} =$ 1 for having history of atrophic gastritis, else 0
$x_{22} =$ 1 for having history of ulcer, else 0
$x_{23} =$ 1 for having history of intestinal metaplasia, else 0
$x_{24} =$ 1 for having history of gastric polyp, else 0
$x_{25} =$ 1 for having history of other gastric diseases, else 0

• The univariate logistic regression model was first applied to choose the appropriate model with each explanatory variable; the history of endoscopy, sex, age group, screening month, hospital type, metropolitan area, history of atrophic gastritis, ulcer, intestinal metaplasia, gastric polyp, and other gastric diseases. Then, using the stepwise selection process at the 0.05 significant level of the score chi-square for entering an effect into the model and the 0.05 significant level for the Wald chi-square for an effect to stay in the model, multivariable logistic regression analysis was performed to assess the adjusted risk factors.

• Analysis of maximum likelihood estimates for multivariable regression model after stepwise selection methods.

< 2013-2014 cycle >

**Parameter**	**DF**	**Estimate**	**SE**	**Wald Chi-square**	***P* value**
Intercept	1	-7.6543	0.0393	37863.82	<.0001
History of endoscopy	1	0.5459	0.0135	1625.518	<.0001
Male	1	0.9966	0.0136	5337.323	<.0001
Age group, 50-59	1	0.8708	0.0229	1449.701	<.0001
Age group, 60-69	1	1.4934	0.0223	4473.743	<.0001
Age group, 70-79	1	2.0514	0.0229	7997.694	<.0001
Age group, 80-	1	2.6717	0.0311	7398.85	<.0001
Month, Feb.	1	-0.0143	0.0412	0.1212	0.7277
Month, Mar.	1	-0.0797	0.0371	4.6039	0.0319
Month, Apr.	1	-0.1295	0.0374	11.9973	0.0005
Month, May	1	-0.1128	0.038	8.819	0.003
Month, Jun.	1	-0.0978	0.0387	6.3872	0.0115
Month, Jul	1	-0.0717	0.0381	3.5417	0.0598
Month, Aug.	1	-0.1133	0.039	8.4305	0.0037
Month, Sep.	1	-0.0832	0.0402	4.2802	0.0386
Month, Oct.	1	-0.1725	0.0376	21.0952	<.0001
Month, Nov.	1	-0.1517	0.0368	16.9715	<.0001

© 2021 Noh, C-K, et al. *JAMA Network Open.*

Month, Dec.	1	-0.2037	0.035	33.9297	<.0001
Metropolitan area	1	-0.1334	0.0128	109.5006	<.0001
History of atrophic gastritis	1	-0.2367	0.028	71.5401	<.0001
History of ulcer	1	-0.1596	0.0301	28.0827	<.0001
History of intestinal metaplasia	1	0.2959	0.0954	9.6217	0.0019
History of gastric polyp	1	0.2367	0.0469	25.4607	<.0001
History of other gastric diseases	1	-0.1988	0.0318	39.0617	<.0001

© 2021 Noh, C-K, et al. *JAMA Network Open.*

< 2015-2016 cycle >

Parameter	DF	Estimate	SE	Wald Chi-square	*P* value
Intercept	1	-7.7296	0.0363	45397.29	<.0001
History of endoscopy	1	0.5292	0.0129	1689.052	<.0001
Male	1	0.9721	0.013	5551.517	<.0001
Age group, 50-59	1	0.9022	0.0223	1640.696	<.0001
Age group, 60-69	1	1.4855	0.0216	4714.973	<.0001
Age group, 70-79	1	2.0223	0.0224	8174.471	<.0001
Age group, 80-	1	2.6381	0.0285	8592.853	<.0001
Month, Feb.	1	-0.0476	0.0366	1.6866	0.194
Month, Mar.	1	-0.0647	0.0319	4.1207	0.0424
Month, Apr.	1	-0.1223	0.0332	13.5901	0.0002
Month, May	1	-0.0926	0.0339	7.4861	0.0062
Month, Jun.	1	-0.0817	0.0356	5.2652	0.0218
Month, Jul	1	-0.0383	0.0344	1.2372	0.266
Month, Aug.	1	-0.083	0.0343	5.8399	0.0157
Month, Sep.	1	-0.0985	0.0352	7.8361	0.0051
Month, Oct.	1	-0.125	0.0324	14.8666	0.0001
Month, Nov.	1	-0.1799	0.0319	31.8317	<.0001
Month, Dec.	1	-0.1811	0.0302	35.9364	<.0001
Hospital	1	0.0209	0.0168	1.5413	0.2144
Clinics	1	-0.0401	0.0138	8.45	0.0037
Metropolitan area	1	0.0209	0.0168	1.5413	0.2144
History of atrophic gastritis	1	-0.0401	0.0138	8.45	0.0037
History of ulcer	1	-0.1356	0.0122	123.382	<.0001
History of intestinal metaplasia	1	-0.2429	0.0255	90.485	<.0001
History of gastric polyp	1	-0.1663	0.0276	36.3501	<.0001
History of other gastric diseases	1	0.3389	0.0746	20.6319	<.0001

• Analyses were performed using SAS version 9.4 (SAS Institute Inc., Cary, NC, USA).
The code for this model is:

© 2021 Noh, C-K, et al. *JAMA Network Open.*

```
*----------- Table 3 ---------;

*------------------------------;
/*logistic regression model */
*------------------------------;

options mprint mlogic;
%let var = hendo sex_type age_gr2 month hospital metropolitan qc_phx_atgs_yn
qc_phx_pu_yn  qc_phx_inmt_yn qc_phx_gpo_yn qc_phx_st_etc;

%macro logistic(ds = );
%let i = 1;
%do %until (%scan(&list., &i.)=);
       %let var = %scan(&list., &i.);
       proc logistic data=&ds.;
              class  hendo(ref='1') sex_type(ref='2') age_gr2(ref='1')
       month(ref='01') hospital (ref='1')metropolitan(ref='0') /param=ref   ;

       model  dc(event='1')=  &var. / scale=none cl details ;
       run;
       %let i= %eval(&i.+1);
%end;
%mend logistic;
%logistic(ds=ed.gj_34_0114); /*univariate, for 2013-2014 cycle*/
%logistic(ds=ed.gj_56_0114); /*univariate, for 2013-2014 cycle*/

/*multiple, for 2013-2014 cycle*/
proc logistic data=ed.gj_34_0114;
       class  hendo(ref='1') sex_type(ref='2') age_gr2(ref='1')
       month(ref='01') hospital (ref='1') metropolitan(ref='0')
       qc_phx_atgs_yn(ref='1') qc_phx_pu_yn(ref='1')  qc_phx_inmt_yn(ref='1')
       qc_phx_gpo_yn(ref='1') qc_phx_st_etc(ref='1') /param=ref   ;
       model  dc(event='1')= hendo sex_type age_gr2 month hospital
       metropolitan qc_phx_atgs_yn qc_phx_pu_yn  qc_phx_inmt_yn qc_phx_gpo_yn
       qc_phx_st_etc  / scale=none cl selection=stepwise details ;
run;

/*multiple, for 2015-2016 cycle*/
proc logistic data=ed.gj_56_0114;
       class  hendo(ref='1') sex_type(ref='2') age_gr2(ref='1')
       month(ref='01') hospital (ref='1') metropolitan(ref='0')
       qc_phx_atgs_yn(ref='1') qc_phx_pu_yn(ref='1')  qc_phx_inmt_yn(ref='1')
       qc_phx_gpo_yn(ref='1') qc_phx_st_etc(ref='1') /param=ref   ;
       model  dc(event='1')= hendo sex_type age_gr2 month hospital
       metropolitan qc_phx_atgs_yn qc_phx_pu_yn  qc_phx_inmt_yn qc_phx_gpo_yn
       qc_phx_st_etc  / scale=none cl selection=stepwise details ;
run;
```

© 2021 Noh, C-K, et al. *JAMA Network Open.*
